# Effect of age and ICU types on mortality in invasive mechanically ventilated patients with sepsis receiving dexmedetomidine: a retrospective cohort study with propensity score matching

**DOI:** 10.3389/fphar.2024.1344327

**Published:** 2024-02-29

**Authors:** Shuai Zhao, Ruihui Zhou, Qi Zhong, Mi Zhang

**Affiliations:** Department of Anesthesiology, Zhongnan Hospital of Wuhan University, Wuhan, China

**Keywords:** dexmedetomidine, invasive mechanical ventilation, sepsis, mortality, elderly patient

## Abstract

**Background:** Dexmedetomidine is recommended for sedation in patients on mechanical ventilation. Whether age or ICU types could alter mortality in invasive mechanically ventilated patients with sepsis receiving dexmedetomidine is unknown.

**Methods:** We included patients with sepsis receiving invasive mechanical ventilation from the Medical Information Mart for Intensive Care IV database. The exposure was intravenous dexmedetomidine administration during ICU stay. The primary outcome was 28-day mortality. The secondary outcomes were the length of ICU stay and liberation from invasive mechanical ventilation. Propensity score matching (PSM) and Cox proportional hazards regression were used to adjust for confounders and investigate any association. Restricted cubic spline models were used to evaluate potential nonlinear associations.

**Results:** The pre-matched and propensity score-matched cohorts included 5,871 and 2016 patients, respectively. In the PSM cohorts, dexmedetomidine exposure was related to lower 28-day mortality (186 [17.7%] *vs*. 319 [30.3%]; *p* < 0.001). Patients receiving dexmedetomidine, regardless of whether they were younger (≤65 years; hazard ratio [HR], 0.31; 95% confidence interval [CI], 0.23–0.42; *p* < 0.001) or elderly (>65 years; HR, 0.65; 95% CI, 0.52–0.83; *p* < 0.001), was associated with lower 28-day mortality (61 [10.3%] *vs.* 168 [28.2%] for younger; 125 [27.2%] *vs.* 152 [33.0%] for elderly). Patients receiving dexmedetomidine was also associated with lower 28-day mortality (53 [12.6%] *vs.* 113 [26.5%] for surgical intensive care unit [SICU]; 133 [21.0%] *vs.* 206 [32.9%] for non-SICU) regardless of whether the first admission to the SICU (HR, 0.36; 95% CI, 0.25–0.50; *p* < 0.001) or non-SICU (HR, 0.50; 95% CI, 0.40–0.62; *p* < 0.001). Moreover, both dose and duration of dexmedetomidine administration were related to lower 28-day mortality than no dexmedetomidine in younger patients (*p* < 0.001), but it not statistically significant in elderly patients.

**Conclusion:** Dexmedetomidine was associated with lower 28-day mortality in critically ill patients with sepsis receiving invasive mechanical ventilation, regardless of whether patients were younger or elderly, the first admission to the SICU or non-SICU.

## 1 Introduction

Sepsis is considered the main cause of increased healthcare costs and in-hospital mortality worldwide. More than 20 million patients with sepsis have severe organ dysfunction, of which at least 20% receive mechanical ventilation (Fleischmann et al., 2016; Dhital et al., 2018). Dexmedetomidine or propofol was recommended for sedation in patients requiring mechanical ventilation (Devlin et al., 2018) to relieve anxiety, enhance comfort, and prevent agitation-related harm.

Dexmedetomidine has been shown to have anti-inflammatory and organ-protective effects superior to those of propofol in animals and humans (Lankadeva et al., 2021; Mei et al., 2021). Dexmedetomidine has also been reported to decrease the infection rate and short-term mortality in patients (Pandharipande et al., 2007; 2010). However, several recent studies have suggested that age, operative status, and ICU types can influence dexmedetomidine’s effects. As a result of a secondary analysis of the SPICE III trial, mechanically ventilated elderly (>65 years) patients treated with dexmedetomidine showed a high probability of reduced 90-day mortality regardless of operative or non-operative status. But younger patients (≤65 years) with non-operative status were at a higher probability of 90-day mortality (Shehabi et al., 2021). Based on the post-hoc analysis of the DESIRE III trial, dexmedetomidine could provide a superior quality of sedation compared to other sedatives in elderly (≥71 years) sepsis patients who require ventilation (Sato et al., 2021). A recent meta-analysis also suggested that age could affect the treatment effects of dexmedetomidine, including the incidence of adverse events and mortality (Heybati et al., 2022). However, no studies have investigated whether age or ICU type affects short-term outcomes in invasive mechanically ventilated patients with sepsis receiving dexmedetomidine.

Therefore, we designed this study to investigate whether age or ICU type could alter 28-day mortality in invasive mechanically ventilated patients with sepsis receiving dexmedetomidine.

## 2 Materials and methods

### 2.1 Study design and ethics approval

This retrospective observational study was based on the Medical Information Mart for Intensive Care IV (MIMIC-IV) (version 2.0) between 2008 and 2019 (Johnson et al., 2022). This database was approved by the Institutional Review Board (IRB) of the Beth Israel Deaconess Medical Center (2001-P-001699/14) and authorized by the Massachusetts Institute of Technology (No. 0403000206) (Johnson et al., 2016). The requirement for written informed consent was waived by the IRB. One author (Shuai Zhao) completed the required training courses of the Collaborative Institutional Training Initiative program and obtained access to the database (certificate number 50055865). This manuscript followed the STrengthening the Reporting of OBservational studies in Epidemiology statement for reporting cohort studies (von Elm et al., 2007).

### 2.2 Patient selection criteria

The inclusion criteria were patients who (1) were admitted to ICU; (2) were diagnosed with sepsis according to the Sepsis-3 criteria, that is, the patient had documented or suspected infection and a Sequential Organ Failure Assessment (SOFA) score ≥ 2 (Singer et al., 2016); and (3) received invasive mechanical ventilation (IMV). The exclusion criteria were patients who (1) aged < 18 years; (2) spent less than 24 h or more than 100 days in the ICU; (3) received IMV < 24 h; and (4) the duration of dexmedetomidine administration was < 24 h. If patients with multiple ICU admissions, only the first was considered.

### 2.3 Exposure and outcomes

The exposure was intravenous dexmedetomidine administration during ICU stay. Patients with a duration of dexmedetomidine administration was < 24 h were excluded. Dexmedetomidine administration was extracted from the prescriptions table. Patients who had missing data regarding dexmedetomidine exposure were excluded in the analysis. The primary outcome was 28-day mortality, defined as death observed within 28-day after ICU admission. The secondary outcomes were length of ICU stay and liberation from IMV.

### 2.4 Data extraction and definitions

The following variables were extracted from the MIMIC-IV database using Structured Query Language (SQL): age, sex, body mass index (BMI), admission type, type of ICU on admission, co-morbidities, laboratory tests, vital signs, SOFA score, Charlson Comorbidities Index (CCI) score, medication information, mechanical ventilation, and renal replacement therapy (RRT). The SQL are widely used for the extraction of data from MIMIC-IV database (Sun et al., 2022; Gu et al., 2023; Ning et al., 2023). The SQL script codes were available from the GitHub website (https://github.com/MIT-LCP/mimic-iv). Laboratory findings, including blood urea nitrogen (BUN) and creatinine, were collected during the first 24 h in ICU. The severity of illness and organ failure was assessed using the SOFA and CCI scores. The value related to the highest severity of illness was used if there were multiple records in the first 24 h. Adverse events within an ICU hospitalization, including hypotension and bradycardia, were identified by the International Classification of Diseases −9 or −10 codes. Medication information on dexmedetomidine and vasopressors during the ICU stay, including doses, routes, rates, and start and end times, was also collected. The duration of IMV was calculated by counting the onset to the closure of ventilator.

### 2.5 Statistical analyses

There was no *a priori* statistical analysis plan because of the retrospective nature of this study. Statistical power calculations were not performed prior to this study, and the sample size was based on available data form the data set. All continuous variables did not meet the normality criteria of distribution and were expressed as median (interquartile range (IQR)). Categorical variables are presented as numbers (%). For between-group comparisons, continuous variables were compared using the Mann-Whitney U-test. Categorical variables were compared using the Chi-squared test or Fisher’s exact test. The missing data of each variable are described in the Supplementary Table S1. Missing data were imputed using multiple imputation (Sterne et al., 2009).

Propensity score matching (PSM) was used to minimize potential confounding factors on selection bias. PSM analysis was based on a logistic regression model using the nearest-neighbor matching method (1:1) with a caliper (0.03).

The variables in the PSM model were chosen based on previous literature, including age, sex, BMI, admission type, type of ICU on admission, co-morbidities, BUN, creatinine, MAP, SOFA score, CCI score, medication use, and RRT treatment. Standardized mean differences (SMD) were calculated to assess the balance between covariates. A SMD of less than 10% after PSM indicated a balance between the two groups. Paired *t*-tests or McNemar tests were used to evaluate significant differences for continuous or categorical variables between the two groups after PSM.

Before and after PSM, Cox proportional hazards regression was performed to estimate the association between dexmedetomidine and 28-day mortality. It was conducted to adjust for confounding variables (age, sex, BMI, admission type, type of ICU on admission, co-morbidities, BUN, creatinine, MAP, SOFA score, CCI score, medication use, and RRT treatment).

Subgroup analysis was conducted before and after PSM to assess whether the association between dexmedetomidine and 28-day mortality varied based on age and ICU type on admission. Before subgroup analysis, the interaction analysis (*P* for interaction) was performed on each subgroup to assess comparability. Moreover, the association between the dose and duration of dexmedetomidine and 28-day mortality was explored after PSM. The nonlinear test was used to determine whether the dose and duration of dexmedetomidine administration had nonlinear effects on 28-day mortality. Restricted cubic spline (RCS) models were used to evaluate potential nonlinear associations between the dose and duration of dexmedetomidine administration and 28-day mortality.

All statistical analyses were performed using STATA software (version 14.0) and R software (version 4.2.2). A *p*-value < 0.05 was considered statistically significant.

## 3 Results

### 3.1 Demographic and clinical information in patients before PSM

**FIGURE 1:**
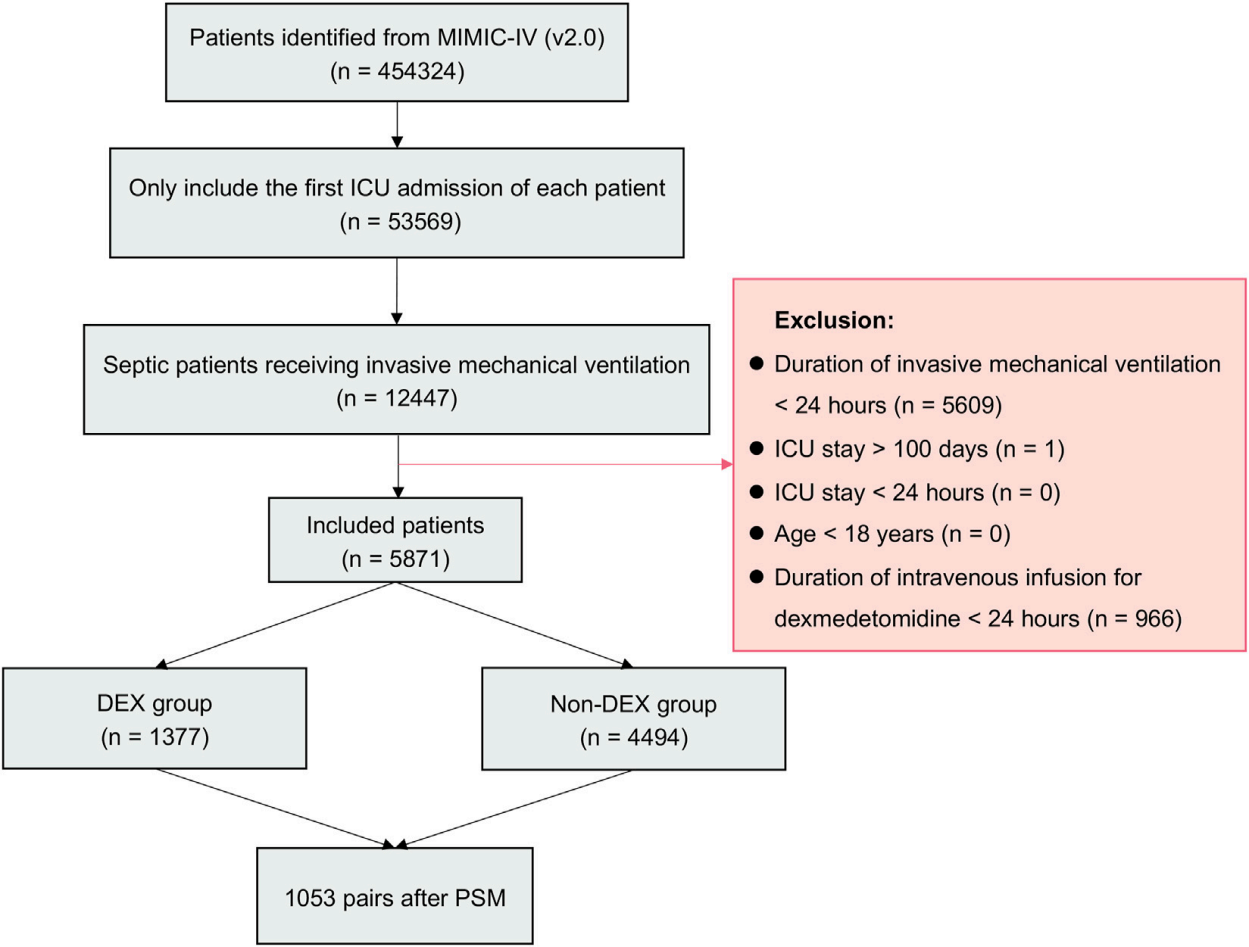
Flowchart of the study. Abbreviations: MIMIC-IV: Medical Information Mart for Intensive Care IV; ICU: intensive care unit; DEX: dexmedetomidine.

From 2008 to 2019, 53,569 ICU admissions were initially identified from MIMIC-IV database. Totally 5,871 patients with sepsis receiving IMV for more than 24 h were included (Figure 1). A total of 1,377 (23.5%) patients received dexmedetomidine (DEX group), while 4494 (76.5%) patients did not receive it (non-DEX group). In the DEX group, 1,374 (99.8%) patients started dexmedetomidine within 24 h after ICU admission. All patients started dexmedetomidine within 48 h after ICU admission. It indicated that this study focused on patients who received dexmedetomidine early after ICU admission. Significant differences were observed in age, sex, BMI, co-morbidities, BUN, MAP, CCI score, and medication use before PSM. The DEX group patients were younger than non-DEX group (*p* < 0.001). The DEX group patients had a higher proportion of male (*p* < 0.001), propofol (*p* < 0.001), and vasopressor use (*p* = 0.003), as well as higher BMI (*p* < 0.001) and MAP (*p* = 0.003) than non-DEX group. The DEX group patients showed a lower proportion of cerebrovascular disease (*p* = 0.043), liver disease (*p* = 0.028), chronic renal disease (*p* = 0.020), tumor (*p* = 0.020), and morphine use (*p* < 0.001) as well as lower BUN (*p* < 0.001) and CCI score (*p* < 0.001) than non-DEX group.

As for clinical outcomes, the DEX group patients showed a lower 28-day mortality (18.2% *vs.* 35.8%; *p* < 0.001), longer length of ICU stay (median 11.8 (IQR 7.6–18.2) *vs.* 6.7 (3.9–12.1) days; *p* < 0.001), and a higher proportion of liberation from IMV (73.7% *vs.* 54.7%; *p* < 0.001) than non-DEX group. Moreover, the DEX group patients showed a lower incidence of bradycardia than non-DEX group (*p* = 0.002); however, the incidence of hypotension was not statistically significant (*p* = 0.626).

### 3.2 Demographic and clinical information in patients after PSM

In PSM, 1,053 pairs of patients between DEX and non-DEX groups were completely matched. In terms of baseline characteristics, no significant differences were observed between the two groups after PSM (Table 1; Supplementary Figure S1). The study groups were equally balanced (SMD < 10%). Similar to the results before PSM, the DEX group patients showed a lower 28-day mortality (17.7% *vs.* 30.3%; *p* < 0.001), longer length of ICU stay (median 11.6 (IQR 7.5–17.6) *vs.* 6.9 (4.2–13.0) days; *p* < 0.001), and a higher proportion of liberation from IMV (74.9% *vs.* 58.1%; *p* < 0.001) than non-DEX group.

**TABLE 1:** Baseline characteristics and outcomes before and after PSM. Values are median (IQR [range]) or number (proportion).

Characteristics	Before PSM (n = 5,871)			After PSM (n = 2106)		
	DEX (n = 1,377)	Non-DEX (n = 4494)	SMD (%)	DEX (n = 1,053)	Non-DEX (n = 1,053)	SMD (%)
Age, median (IQR), year	61.0 (49.2–72.6)	66.6 (54.7–77.8)	−28.8	61.9 (49.9–72.9)	62.2 (49.4–74.5)	2.6
>65 years, n (%)	574 (41.7)	2403 (53.5)		459 (43.6)	460 (43.7)	
Sex, male, n (%)	871 (63.3)	2461 (54.8)	17.3	647 (61.4)	644 (61.2)	0.6
BMI, median (IQR), kg/m^2^	28.9 (24.8–34.5)	28.0 (24.5–32.2)	16.0	28.9 (24.7–34.1)	28.0 (25.2–33.8)	−7.3
Admission type, n (%)			−3.5			1.0
Elective	24 (1.7)	100 (2.2)		21 (2.0)	18 (1.7)	
Non-elective	1,353 (98.3)	4394 (97.8)		1,032 (98.0)	1,035 (98.3)	
Type of ICU on admission, n (%)			5.4			−3.3
SICU	544 (39.5)	1,658 (36.9)		429 (39.8)	427 (40.6)	
Non-SICU	833 (60.5)	2836 (63.1)		634 (60.2)	626 (59.4)	
Co-morbidities, n(%)						
Congestive heart failure	408 (29.6)	1,332 (29.6)	0	305 (29.0)	288 (27.4)	6.5
Cerebrovascular disease	241 (17.5)	899 (20.0)	−6.4	188 (17.9)	190 (18.0)	1.3
Chronic pulmonary disease	410 (29.8)	1,265 (28.1)	3.6	310 (29.4)	311 (29.5)	−0.3
Liver disease	235 (17.1)	886 (19.7)	−6.8	179 (17.0)	177 (16.8)	−1.3
Diabetes	385 (28.0)	1,298 (28.9)	−2.0	293 (27.8)	283 (26.9)	4.0
Chronic renal disease	239 (17.4)	909 (20.2)	−7.4	180 (17.1)	157 (14.9)	6.0
Tumor	155 (11.3)	614 (13.7)	−7.3	113 (10.7)	128 (12.2)	−1.1
Laboratory tests, median (IQR)						
BUN, mg/dL	23 (16–36)	25 (17–42)	−13.6	23 (16–38)	23 (15–36)	2.7
Creatinine, mg/dL	1.2 (0.9–1.9)	1.2 (0.9–2.1)	−6.4	1.2 (0.9–2.0)	1.2 (0.8–1.8)	3.7
MAP, median (IQR), mmHg	78.2 (73.6–84.8)	77.6 (72.1–84.9)	7.2	78.0 (73.2–84.7)	78.3 (73.3–86.1)	−4.9
Severity of illness, median (IQR)						
SOFA score	9 (6–12)	9 (6–12)	1.0	9 (6–12)	8 (6–11)	3.8
CCI score	5 (3–7)	6 (4–8)	−19.6	5 (3–7)	5 (3–7)	5.2
Medications, n (%)						
Propofol	1,292 (93.8)	3463 (77.1)	48.9	976 (92.7)	976 (92.7)	−0.6
Midazolam	788 (57.2)	2544 (56.6)	1.2	578 (54.9)	587 (55.7)	2.8
Morphine	302 (21.9)	1,365 (30.4)	−19.3	245 (23.3)	258 (24.5)	−3.8
Vasopressors	1,061 (77.1)	3279 (73.0)	9.4	793 (75.3)	788 (74.8)	3.7
RRT, n (%)	103 (7.5)	364 (8.1)	−2.3	78 (7.4)	65 (6.2)	1.9
Outcomes						
28-day mortality, n (%)	250 (18.2)	1,607 (35.8)		186 (17.7)	319 (30.3)	
Length of ICU stay, median (IQR)	11.8 (7.6–18.2)	6.7 (3.9–12.1)		11.6 (7.5–17.6)	6.9 (4.2–13.0)	
Liberation from IMV, n (%)	1,015 (73.7)	2457 (54.7)		789 (74.9)	612 (58.1)	
Adverse events, n (%)						
Hypotension	67 (4.9)	235 (5.2)		44 (4.2)	52 (4.9)	
Bradycardia	39 (2.8)	66 (1.5)		24 (2.3)	13 (1.2)	

Abbreviations: PSM: propensity score matching; DEX: dexmedetomidine; SMD: standardized mean difference; IQR: interquartile range; BMI: body mass index; BUN: blood urea nitrogen; MAP: mean arterial pressure; SOFA: sequential organ failure assessment; CCI: charlson comorbidities index; RRT: renal replacement therapy; ICU: intensive care unit; SICU: surgical intensive care unit; IMV: invasive mechanical ventilation.

### 3.3 Association between dexmedetomidine and 28-day mortality

**FIGURE 2:**
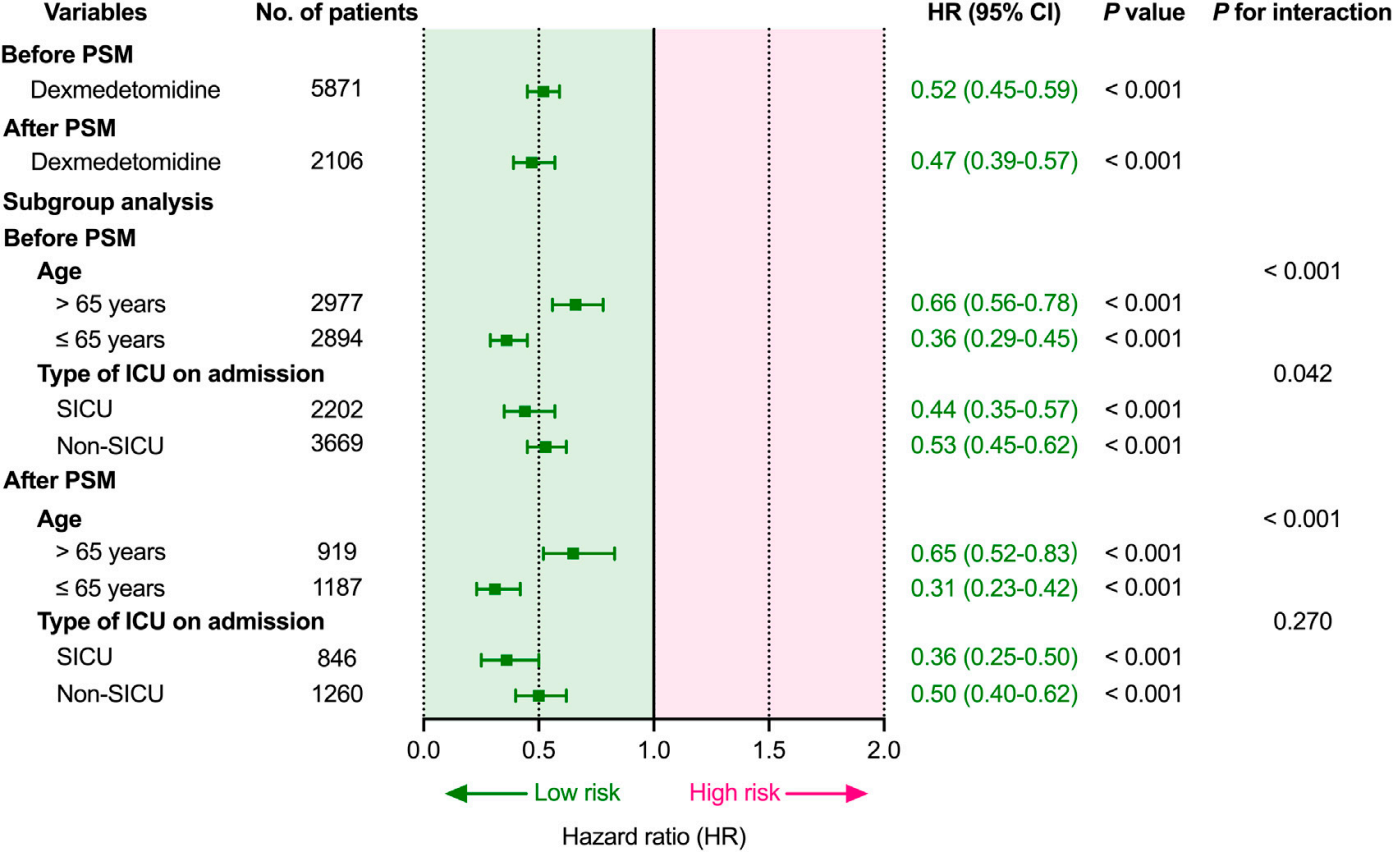
Association between dexmedetomidine and 28-day mortality and subgroup analysis. Abbreviations: HR: hazard ratio; CI: confidence interval; PSM: propensity score matching; ICU: intensive care unit; SICU: surgical intensive care unit.

Cox proportional hazards regression were used to estimate the association between dexmedetomidine and 28-day mortality. Before PSM, dexmedetomidine was related to a lower 28-day mortality (HR 0.52, 95%CI (0.45–0.59); *p* < 0.001) after adjusting for possible confounding factors (Figure 2; Supplementary Table S2). Similar to the results before PSM, dexmedetomidine was also related to lower 28-day mortality (HR 0.47, 95%CI (0.39–0.57); *p* < 0.001) (Figure 2; Supplementary Table S3) in the population after PSM.

### 3.4 Subgroup analysis for age and type of ICU on admission

Before subgroup analysis, interaction effects were evaluated between age and dexmedetomidine exposure (*P* for interaction < 0.001; < 0.001) and between the type of ICU on admission and dexmedetomidine exposure (*P* for interaction = 0.042; = 0.270) before and after PSM (Figure 2). Dexmedetomidine was related to lower 28-day mortality in all subgroups before and after PSM. Dexmedetomidine was related to lower 28-day mortality, regardless of whether the patients were younger (≤65 years; *p* < 0.001) or elderly (>65 years; *p* < 0.001). Moreover, dexmedetomidine was also related to lower 28-day mortality, regardless of first admission was SICU (*p* < 0.001) or non-SICU (*p* < 0.001).

### 3.5 Dose- and duration-response relationship between dexmedetomidine and 28-day mortality

**FIGURE 3:**
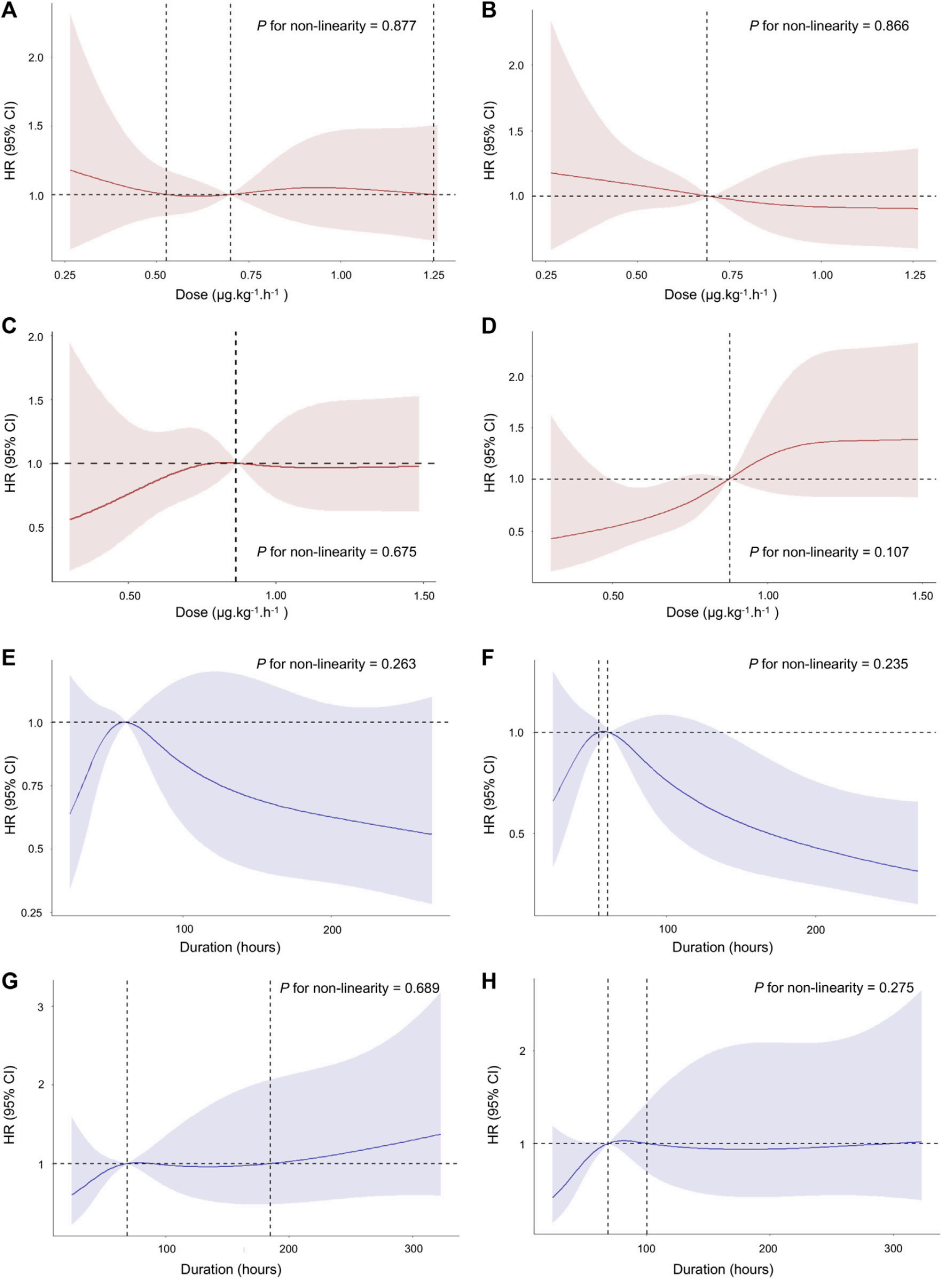
Association between the dose and duration of dexmedetomidine administration and 28-day mortality using RCS. The curve of HR and dose rate using univariate **(A)** and multivariate **(B)** Cox regression with RCS in age > 65 years. The curve of HR and dose rate using univariate **(C)** and multivariate **(D)** Cox regression with RCS in age ≤ 65 years. The curve of HR and duration using univariate **(E)** and multivariate **(F)** Cox regression with RCS in age > 65 years. The curve of HR and duration using univariate **(G)** and multivariate **(H)** Cox regression with RCS in age ≤ 65 years. The multivariate Cox regression adjusted for age, gender, BMI, admission type, type of ICU on admission, co-morbidities, BUN, creatinine, MAP, SOFA score, CCI score, medication use, and RRT treatment. Cubic spline curves are shown as a solid line, with the shaded area representing the 95% confidence intervals. Abbreviations: RCS: restricted cubic spline; HR: hazard ratio; BUN: blood urea nitrogen; MAP: mean arterial pressure; SOFA: Sequential Organ Failure Assessment; CCI: Charlson Comorbidities Index; RRT: renal replacement therapy.

After PSM, the median dose rate of dexmedetomidine administration was 0.81 (IQR, 0.57–1.01) μg.kg^-1^. h^-1^, and the median duration of dexmedetomidine administration was 67 (IQR, 41–113) hours. Considering the dose and duration as continuous variables, we firstly performed the nonlinear test to examine whether the dose and duration of dexmedetomidine administration had nonlinear effects on 28-day mortality. However, the nonlinear test indicated that the relationship between the dose and duration of dexmedetomidine administration and 28-day mortality was not significantly nonlinear (*p* > 0.05) (Figure 3). The results of univariable or multivariable Cox regression with RCS also suggested that the dose and duration thresholds regarding 28-day mortality reduction could not be calculated (Figure 3).

**FIGURE 4:**
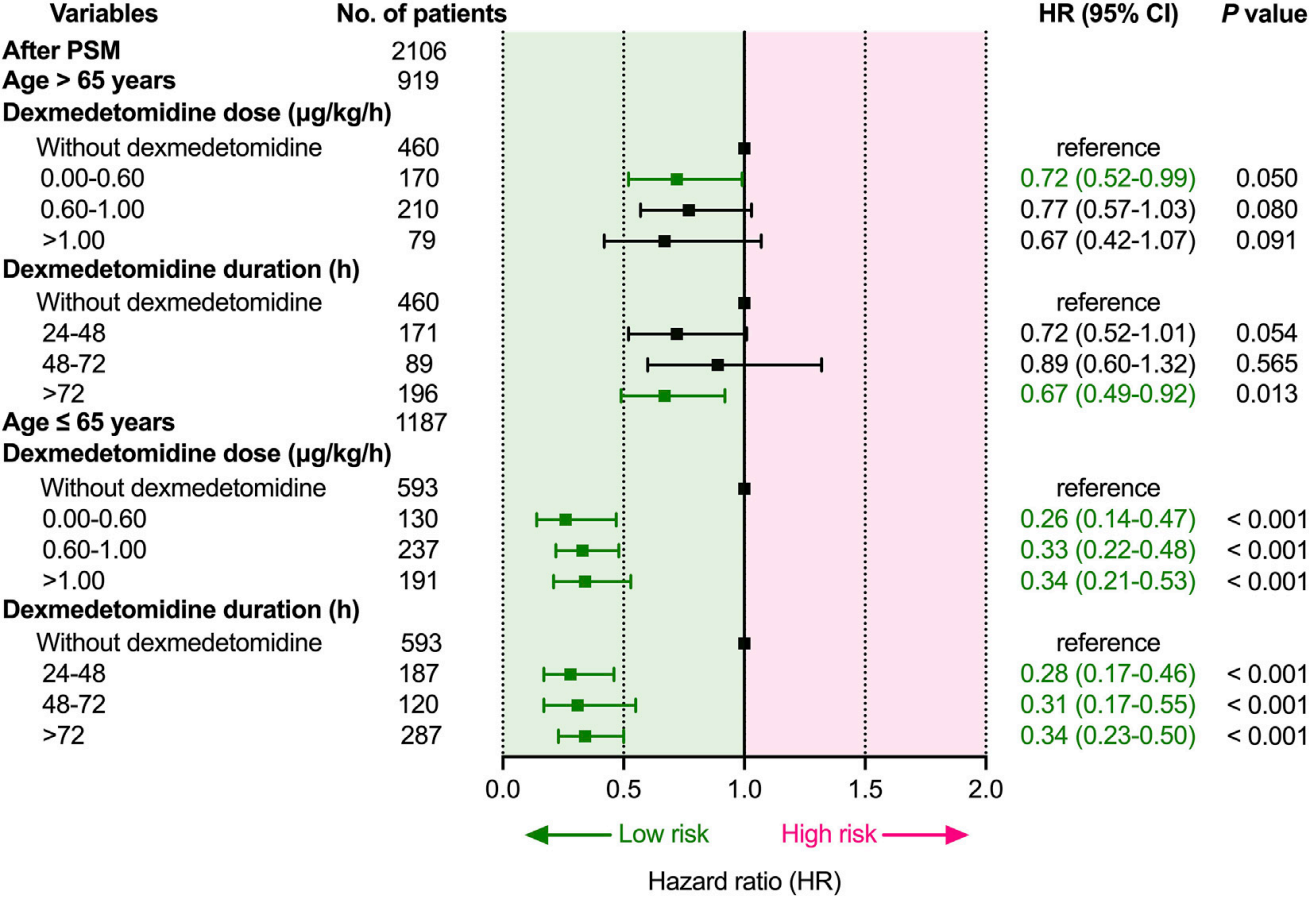
Dose- and duration-response relationship between dexmedetomidine and 28-day mortality. Abbreviations: HR: hazard ratio; CI: confidence interval; PSM: propensity score matching.

Subsequently, we converted dose and duration to categorical variables based on a previous study for further analysis (Hu et al., 2022). As shown in Figure 4, in elderly patients (>65 years), the duration of dexmedetomidine > 72 h was related to lower 28-day mortality than non-DEX (*p* = 0.013). However, the relationship between the dose rate of dexmedetomidine and 28-day mortality was not statistically different. Moreover, in younger patients (≤65 years), both the dose rate and duration of dexmedetomidine were related to lower 28-day mortality (*p* < 0.001).

## 4 Discussion

This study suggested that dexmedetomidine was related to lower 28-day mortality, regardless of whether patients were younger or elderly, first admission to SICU or non-SICU in patients with sepsis receiving IMV. Our study also revealed that both the dose and duration of dexmedetomidine administration were related to lower 28-day mortality than no dexmedetomidine in younger patients. However, this relationship was not statistically significant in elderly patients.

Dexmedetomidine is recommended for sedation in patients requiring mechanical ventilation; however, whether dexmedetomidine is a better sedative for patients with sepsis requiring IMV remains a matter of debate. Our results suggested that dexmedetomidine administration was related to lower 28-day mortality, longer length of ICU stay, and a higher proportion of liberation from IMV than no dexmedetomidine treatment. Cox proportional hazards regression results also supported that dexmedetomidine was related to lower 28-day mortality. These results were consistent with previous studies (Song et al., 2019; Aso et al., 2021; Zhang et al., 2022). However, the DESIRE trial indicated that dexmedetomidine treatment did not improve 28-day mortality in patients requiring mechanical ventilation with sepsis (Kawazoe et al., 2017). This difference may be due to the following reasons. First, this study included patients with sepsis who required invasive and noninvasive ventilation. Our study only included patients with a higher severity of invasive mechanical ventilation. Second, this study had in fact identified the clinically important benefits of dexmedetomidine. The risk of 28-day mortality was reduced by 8% with dexmedetomidine, but the difference was not statistically significant (22.8% *vs.* 30.8%; n = 202; *p* = 0.200). Our study has sufficient statistical power to detect differences in mortality (18.2% *vs.* 35.8%; *p* < 0.001; n = 5,871; 17.7% *vs.* 30.3%; *p* < 0.001; n = 2106). Nevertheless, our study provides important information that dexmedetomidine administration remains significant in reducing the 28-day mortality. There is a need for further randomized controlled trials to confirm these findings.

Importantly, several studies have found that age or ICU type could influence the clinical effects of dexmedetomidine. As a result of a secondary analysis of the SPICE III trial, mechanically ventilated elderly (>65 years) patients treated with dexmedetomidine showed a high probability of reduced 90-day mortality regardless of operative or non-operative status. But younger patients (≤65 years) with non-operative status were at a higher probability of 90-day mortality (Shehabi et al., 2021). A recent meta-analysis also suggested that age could affect the treatment effects of dexmedetomidine, including the incidence of adverse events and mortality (Heybati et al., 2022). However, our study suggests that dexmedetomidine administration is related to lower 28-day mortality in both younger and elderly patients. The reason for these discrepancies might be the different study subjects (septic patients requiring IMV *vs*. critically ill adults requiring mechanical ventilation) and follow-up time points (28-day *vs.* 90-day). The patients in our study represent a sample of more severely ill patients, they were diagnosed with sepsis requiring IMV. In addition, previous studies have reported that no significant differences were found in the length of ICU stay, duration of mechanical ventilation, and cardiovascular adverse events associated with dexmedetomidine between the different ICU types (Pereira et al., 2020; Abowali et al., 2021; Heybati et al., 2022). We hypothesized that non-SICU patients may have other underlying comorbidities that influence the effects of dexmedetomidine. However, our results suggested that dexmedetomidine was related to lower 28-day mortality, regardless of whether patient was admitted to the SICU or non-SICU. To our knowledge, no study has reported the effects of ICU type on 28-day mortality in patients. Prospective studies are required to investigate and validate the effects of age and ICU type separately on clinical outcomes in this population.

Our study further investigated the dose- and duration-response relationship between dexmedetomidine and 28-day mortality. Although the relationship curves appeared to be nonlinear, it was not statistically significant. Notably, the curves of HR and duration using univariate and multivariate Cox regression with RCS were almost less than 1 in both younger and elderly. Subsequently, we converted dose and duration to categorical variables for further analysis, our results suggested that both the dose and duration of dexmedetomidine administration were associated with lower 28-day mortality than no dexmedetomidine treatment in younger patients. There also appears to be a benefit for elderly patients. However, this difference was also not statistically significant. The results were consistent in both analyses. These differences were not statistically significant, possibly because of the small sample size after PSM (n = 2106). However, to our knowledge, this is the largest reported sample of patients to investigate the effect of age and ICU types on short-term outcomes in this population so far. Future randomized controlled trials are required to determine the optimal dosing regimen of dexmedetomidine in this population. Moreover, a study suggested that both the dose and duration of dexmedetomidine were related to a decreased risk of mortality compared with no dexmedetomidine in sepsis-associated acute kidney injury (Hu et al., 2022). However, no previous studies have reported an association between the dose and duration of dexmedetomidine with 28-day mortality in critically ill patients with sepsis requiring IMV and the potential effects of age on this association. The present study might indicate that the younger patients had even greater treatment benefits with dexmedetomidine than elderly patients. The pharmacokinetic and pharmacodynamic characteristics of dexmedetomidine differ between younger and elderly patients (Weerink et al., 2017; Xu et al., 2017), which may help to interpret these results.

Our study also has several limitations. First, owing to the constraints of a retrospective study based on a public database, information about the strategies for sedation and sedation goals was lacking. Dexmedetomidine administration practices may not be uniform. In order to minimize effect modification due to different dexmedetomidine regimens used among patients, we included only patients who received the duration of dexmedetomidine administration was ≥ 24 h. All patients started dexmedetomidine within 48 h after ICU admission. We also performed subgroup and nonlinear analysis and demonstrated consistency in the results. Second, although we controlled for several potential confounders using PSM and multivariate Cox proportional hazards regression analysis, residual confounding factors may be possible. Third, a retrospective study can only establish an association but not causation. Future randomized controlled trials are required to determine which patients would benefit most from dexmedetomidine and its optimal dosing regimen. Fourth, there could be a difference in sepsis patient care practices between dexmedetomidine administration and not. Patients with dexmedetomidine may have improved mobility. These factors potentially influence outcomes. But this is not the main purpose of this study, it deserves further investigation.

## 5 Conclusion

Among patients with sepsis requiring IMV, dexmedetomidine administration was related to lower 28-day mortality, regardless of whether patients were younger or elderly, or first admission to the SICU or non-SICU. Moreover, the dose and duration of dexmedetomidine administration were both associated with lower 28-day mortality than no dexmedetomidine treatment in younger patients. However, this difference was not statistically significant in elderly patients. These findings should be confirmed by independent randomized controlled trials in the future.

## Data Availability

The original contributions presented in the study are included in the article/Supplementary Material, further inquiries can be directed to the corresponding authors.

## References

[B1] Abowali H. A. Paganini M. Enten G. Elbadawi A. Camporesi E. M. (2021). Critical review and meta-analysis of postoperative sedation after adult cardiac surgery: dexmedetomidine versus propofol. J. Cardiothorac. Vasc. Anesth. 35, 1134–1142. 10.1053/j.jvca.2020.10.022 33168430

[B2] Aso S. Matsui H. Fushimi K. Yasunaga H. (2021). Dexmedetomidine and mortality from sepsis requiring mechanical ventilation: a Japanese nationwide retrospective cohort study. J. Intensive Care Med. 36, 1036–1043. 10.1177/0885066620942154 32696714

[B3] Devlin J. W. Skrobik Y. Gélinas C. Needham D. M. Slooter A. J. C. Pandharipande P. P. (2018). Clinical practice guidelines for the prevention and management of Pain, agitation/sedation, delirium, immobility, and sleep disruption in adult patients in the ICU. Crit. Care Med. 46, e825–e873. 10.1097/CCM.0000000000003299 30113379

[B4] Dhital R. Basnet S. Poudel D. R. (2018). Predictors and outcome of invasive mechanical ventilation in hospitalized patients with sepsis: data from National Inpatient Sample. J. community Hosp. Intern. Med. Perspect. 8, 49–52. 10.1080/20009666.2018.1450592 29686786 PMC5906765

[B5] Fleischmann C. Scherag A. Adhikari N. K. J. Hartog C. S. Tsaganos T. Schlattmann P. (2016). Assessment of global incidence and mortality of hospital-treated sepsis. Current estimates and limitations. Am. J. Respir. Crit. Care Med. 193, 259–272. 10.1164/rccm.201504-0781OC 26414292

[B6] Gu W.-J. Duan X.-J. Liu X.-Z. Cen Y. Tao L.-Y. Lyu J. (2023). Association of magnesium sulfate use with mortality in critically ill patients with sepsis: a retrospective propensity score-matched cohort study. Br. J. Anaesth. 131, 861–870. 10.1016/j.bja.2023.08.005 37684164

[B7] Heybati K. Zhou F. Ali S. Deng J. Mohananey D. Villablanca P. (2022). Outcomes of dexmedetomidine versus propofol sedation in critically ill adults requiring mechanical ventilation: a systematic review and meta-analysis of randomised controlled trials. Br. J. Anaesth. 129, 515–526. 10.1016/j.bja.2022.06.020 35961815

[B8] Hu H. An S. Sha T. Wu F. Jin Y. Li L. (2022). Association between dexmedetomidine administration and outcomes in critically ill patients with sepsis-associated acute kidney injury. J. Clin. Anesth. 83, 110960. 10.1016/j.jclinane.2022.110960 36272399

[B9] Johnson A. Bulgarelli L. Pollard T. Horng S. Celi L. A. Mark R. (2022). MIMIC-IV (version 2.0). PhysioNet. 10.13026/7vcr-e114

[B10] Johnson A. E. W. Pollard T. J. Shen L. Lehman L.-W. H. Feng M. Ghassemi M. (2016). MIMIC-III, a freely accessible critical care database. Sci. data 3, 160035. 10.1038/sdata.2016.35 27219127 PMC4878278

[B11] Kawazoe Y. Miyamoto K. Morimoto T. Yamamoto T. Fuke A. Hashimoto A. (2017). Effect of dexmedetomidine on mortality and ventilator-free days in patients requiring mechanical ventilation with sepsis: a randomized clinical trial. JAMA 317, 1321–1328. 10.1001/jama.2017.2088 28322414 PMC5469298

[B12] Lankadeva Y. R. Shehabi Y. Deane A. M. Plummer M. P. Bellomo R. May C. N. (2021). Emerging benefits and drawbacks of α(2) -adrenoceptor agonists in the management of sepsis and critical illness. Br. J. Pharmacol. 178, 1407–1425. 10.1111/bph.15363 33450087

[B13] Mei B. Li J. Zuo Z. (2021). Dexmedetomidine attenuates sepsis-associated inflammation and encephalopathy via central α2A adrenoceptor. Brain. Behav. Immun. 91, 296–314. 10.1016/j.bbi.2020.10.008 33039659 PMC7749843

[B14] Ning Y.-L. Sun C. Xu X.-H. Li L. Ke Y.-J. Mai Y. (2023). Tendency of dynamic vasoactive and inotropic medications data as a robust predictor of mortality in patients with septic shock: an analysis of the MIMIC-IV database. Front. Cardiovasc. Med. 10, 1126888. 10.3389/fcvm.2023.1126888 37082452 PMC10112491

[B15] Pandharipande P. P. Pun B. T. Herr D. L. Maze M. Girard T. D. Miller R. R. (2007). Effect of sedation with dexmedetomidine vs lorazepam on acute brain dysfunction in mechanically ventilated patients: the MENDS randomized controlled trial. JAMA 298, 2644–2653. 10.1001/jama.298.22.2644 18073360

[B16] Pandharipande P. P. Sanders R. D. Girard T. D. McGrane S. Thompson J. L. Shintani A. K. (2010). Effect of dexmedetomidine versus lorazepam on outcome in patients with sepsis: an *a priori*-designed analysis of the MENDS randomized controlled trial. Crit. Care 14, R38. 10.1186/cc8916 20233428 PMC2887145

[B17] Pereira J. V. Sanjanwala R. M. Mohammed M. K. Le M.-L. Arora R. C. (2020). Dexmedetomidine versus propofol sedation in reducing delirium among older adults in the ICU: a systematic review and meta-analysis. Eur. J. Anaesthesiol. 37, 121–131. 10.1097/EJA.0000000000001131 31860605

[B18] Sato T. Kawazoe Y. Miyagawa N. Yokokawa Y. Kushimoto S. Miyamoto K. (2021). Effect of age on dexmedetomidine treatment for ventilated patients with sepsis: a post-hoc analysis of the DESIRE trial. Acute Med. Surg. 8, e644. 10.1002/ams2.644 33859826 PMC8033411

[B19] Shehabi Y. Serpa Neto A. Howe B. D. Bellomo R. Arabi Y. M. Bailey M. (2021). Early sedation with dexmedetomidine in ventilated critically ill patients and heterogeneity of treatment effect in the SPICE III randomised controlled trial. Intensive Care Med. 47, 455–466. 10.1007/s00134-021-06356-8 33686482 PMC7939103

[B20] Singer M. Deutschman C. S. Seymour C. W. Shankar-Hari M. Annane D. Bauer M. (2016). The third international consensus definitions for sepsis and septic shock (Sepsis-3). JAMA 315, 801–810. 10.1001/jama.2016.0287 26903338 PMC4968574

[B21] Song Y. Gao S. Tan W. Qiu Z. Zhou H. Zhao Y. (2019). Dexmedetomidine versus midazolam and propofol for sedation in critically ill patients: mining the Medical Information Mart for Intensive Care data. Ann. Transl. Med. 7, 197. 10.21037/atm.2019.04.14 31205915 PMC6545304

[B22] Sterne J. A. C. White I. R. Carlin J. B. Spratt M. Royston P. Kenward M. G. (2009). Multiple imputation for missing data in epidemiological and clinical research: potential and pitfalls. BMJ 338, b2393. 10.1136/bmj.b2393 19564179 PMC2714692

[B23] Sun W. Yan Y. Hu S. Liu B. Wang S. Yu W. (2022). The effects of midazolam or propofol plus fentanyl on ICU mortality: a retrospective study based on the MIMIC-IV database. Ann. Transl. Med. 10, 219. 10.21037/atm-22-477 35280412 PMC8908132

[B24] von Elm E. Altman D. G. Egger M. Pocock S. J. Gøtzsche P. C. Vandenbroucke J. P. (2007). The Strengthening the Reporting of Observational Studies in Epidemiology (STROBE) statement: guidelines for reporting observational studies. PLoS Med. 4, e296. 10.1371/journal.pmed.0040296 17941714 PMC2020495

[B25] Weerink M. A. S. Struys M. M. R. F. Hannivoort L. N. Barends C. R. M. Absalom A. R. Colin P. (2017). Clinical pharmacokinetics and pharmacodynamics of dexmedetomidine. Clin. Pharmacokinet. 56, 893–913. 10.1007/s40262-017-0507-7 28105598 PMC5511603

[B26] Xu B. Li Z. Zhou D. Li L. Li P. Huang H. (2017). The influence of age on sensitivity to dexmedetomidine sedation during spinal anesthesia in lower limb orthopedic surgery. Anesth. Analg. 125, 1907–1910. 10.1213/ANE.0000000000002531 28991112

[B27] Zhang T. Mei Q. Dai S. Liu Y. Zhu H. (2022). Use of dexmedetomidine in patients with sepsis: a systematic review and meta-analysis of randomized-controlled trials. Ann. Intensive Care 12, 81. 10.1186/s13613-022-01052-2 36029410 PMC9420168

