# Supplementary material for: Effect of age and ICU types on mortality in invasive mechanically ventilated patients with sepsis receiving dexmedetomidine: a retrospective cohort study with propensity score matching
Source: Front Pharmacol. 2024 Feb 29;15:1344327. doi: 10.3389/fphar.2024.1344327 (PMC10937464; doi:10.3389/fphar.2024.1344327)
Supplement: Supplementary file 1 [file Table1.DOCX]

**Supplementary Materials**

**Table S1.** Percentage on missing data of each variable.

**Table S2.** Multivariable Cox regression analysis for 28-day mortality before PSM (n = 5871).

**Table S3.** Multivariable Cox regression analysis for 28-day mortality of patients after PSM (n = 2106).

**Figure S1.** The standardized mean difference of variables before and after PSM.

**Table S1.** Percentage on missing data of each variable.

| **Variables** | **Missing count** | **Missing rate (%)** |
| --- | --- | --- |
| Age | 0 | 0.00 |
| Sex | 0 | 0.00 |
| **BMI** | **637** | **10.85** |
| Admission type | 0 | 0.00 |
| Type of ICU on admission | 0 | 0.00 |
| Congestive heart failure | 0 | 0.00 |
| Cerebrovascular disease | 0 | 0.00 |
| Chronic pulmonary disease | 0 | 0.00 |
| Liver disease | 0 | 0.00 |
| Diabetes | 0 | 0.00 |
| Chronic renal disease | 0 | 0.00 |
| Tumor | 0 | 0.00 |
| **BUN** | **9** | **0.15** |
| **Creatinine** | **8** | **0.14** |
| MAP | 40 | 0.68 |
| SOFA score | 0 | 0.00 |
| CCI score | 0 | 0.00 |
| Propofol | 0 | 0.00 |
| Midazolam | 0 | 0.00 |
| Morphine | 0 | 0.00 |
| Vasopressors | 0 | 0.00 |
| RRT | 0 | 0.00 |
| 28-day mortality | 0 | 0.00 |
| Length of ICU stay | 0 | 0.00 |
| Liberation from IMV | 0 | 0.00 |
| Hypotension | 0 | 0.00 |
| Bradycardia | 0 | 0.00 |

Abbreviations: PSM: propensity score matching; CI: confidence interval; BMI: body mass index; BUN: blood urea nitrogen; MAP: mean artery pressure; SOFA: Sequential Organ Failure Assessment; CCI: Charlson Comorbidities Index; RRT: Renal replacement therapy; ICU: intensive care unit; SICU: surgical intensive care unit; IMV: invasive mechanical ventilation

**Table S2.** Multivariable Cox regression analysis for 28-day mortality of patients with sepsis receiving invasive mechanical ventilation before PSM (n = 5871).

| **Characteristics** | **HR (95% CI)** | ***P* value** |
| --- | --- | --- |
| Age > 65 years | 1.21 (1.07-1.36) | 0.002 |
| Sex (male vs female) | 1.03 (0.94-1.13) | 0.500 |
| BMI | 0.99 (0.98-1.00) | 0.010 |
| Admission type (Elective vs Non-elective) | 0.33 (0.20-0.52) | <0.001 |
| Type of ICU on admission (SICU vs Non-SICU) | 1.01 (0.91-1.13) | 0.810 |
| **Co-morbidities** |  |  |
| Congestive heart failure | 0.91 (0.81-1.02) | 0.093 |
| Cerebrovascular disease | 1.34 (1.19-1.52) | <0.001 |
| Chronic pulmonary disease | 0.92 (0.83-1.03) | 0.144 |
| Liver disease | 1.16 (1.02-1.32) | 0.020 |
| Diabetes | 0.84 (0.75-0.95) | 0.004 |
| Chronic renal disease | 0.85 (0.74-0.99) | 0.038 |
| Tumor | 1.18 (1.00-1.39) | 0.044 |
| **Laboratory tests** |  |  |
| BUN | 1.00 (1.00-1.01) | 0.016 |
| Creatinine | 0.97 (0.93-1.01) | 0.153 |
| MAP | 1.01 (1.01-1.02) | <0.001 |
| **Severity of illness** |  |  |
| SOFA score | 1.08 (1.07-1.10) | <0.001 |
| CCI score | 1.06 (1.03-1.10) | <0.001 |
| **Medications** |  |  |
| Dexmedetomidine | 0.52 (0.45-0.59) | <0.001 |
| Propofol | 0.49 (0.44-0.55) | <0.001 |
| Midazolam | 0.78 (0.70-0.86) | <0.001 |
| Morphine | 2.09 (1.90-2.29) | <0.001 |
| Vasopressors | 1.75 (1.52-2.01) | <0.001 |
| RRT | 1.27 (1.07-1.52) | 0.006 |

Abbreviations: PSM: propensity score matching; CI: confidence interval; BMI: body mass index; BUN: blood urea nitrogen; MAP: mean artery pressure; SOFA: Sequential Organ Failure Assessment; CCI: Charlson Comorbidities Index; RRT: Renal replacement therapy; ICU: intensive care unit; SICU: surgical intensive care unit

**Table S3.** Multivariable Cox regression analysis for 28-day mortality of patients with sepsis receiving invasive mechanical ventilation after PSM (n = 2106).

| **Characteristics** | **HR (95% CI)** | ***P* value** |
| --- | --- | --- |
| Age > 65 years | 1.29 (1.03-1.61) | 0.025 |
| Sex (male vs female) | 0.82 (0.68-0.98) | 0.027 |
| BMI | 0.98 (0.97-1.00) | 0.010 |
| Admission type (Elective vs Non-elective) | 0.38 (0.16-0.93) | 0.035 |
| Type of ICU on admission (SICU vs Non-SICU) | 0.95 (0.78-1.17) | 0.653 |
| **Co-morbidities** |  |  |
| Congestive heart failure | 1.00 (0.81-1.24) | 0.994 |
| Cerebrovascular disease | 1.37 (1.07-1.75) | 0.013 |
| Chronic pulmonary disease | 0.98 (0.80-1.21) | 0.878 |
| Liver disease | 1.30 (1.00-1.68) | 0.047 |
| Diabetes | 0.77 (0.6100.96) | 0.023 |
| Chronic renal disease | 0.86 (0.64-1.16) | 0.328 |
| Tumor | 1.32 (0.97-1.79) | 0.080 |
| **Laboratory tests** |  |  |
| BUN | 1.00 (1.00-1.01) | 0.377 |
| Creatinine | 0.97 (0.89-1.05) | 0.409 |
| MAP | 1.02 (1.01-1.03) | 0.001 |
| **Severity of illness** |  |  |
| SOFA score | 1.08 (1.05-1.11) | <0.001 |
| CCI score | 1.08 (1.02-1.14) | 0.013 |
| **Medications** |  |  |
| Dexmedetomidine | 0.47 (0.39-0.57) | <0.001 |
| Propofol | 0.79 (0.57-1.08) | 0.133 |
| Midazolam | 0.92 (0.76-1.10) | 0.360 |
| Morphine | 2.46 (2.05-2.96) | <0.001 |
| Vasopressors | 2.34 (1.72-3.18) | <0.001 |
| RRT | 1.75 (1.23-2.48) | 0.002 |

Abbreviations: PSM: propensity score matching; CI: confidence interval; BMI: body mass index; BUN: blood urea nitrogen; MAP: mean artery pressure; SOFA: Sequential Organ Failure Assessment; CCI: Charlson Comorbidities Index; RRT: Renal replacement therapy; ICU: intensive care unit; SICU: surgical intensive care unit

**Figure S1.** The standardized mean difference of variables before and after PSM.


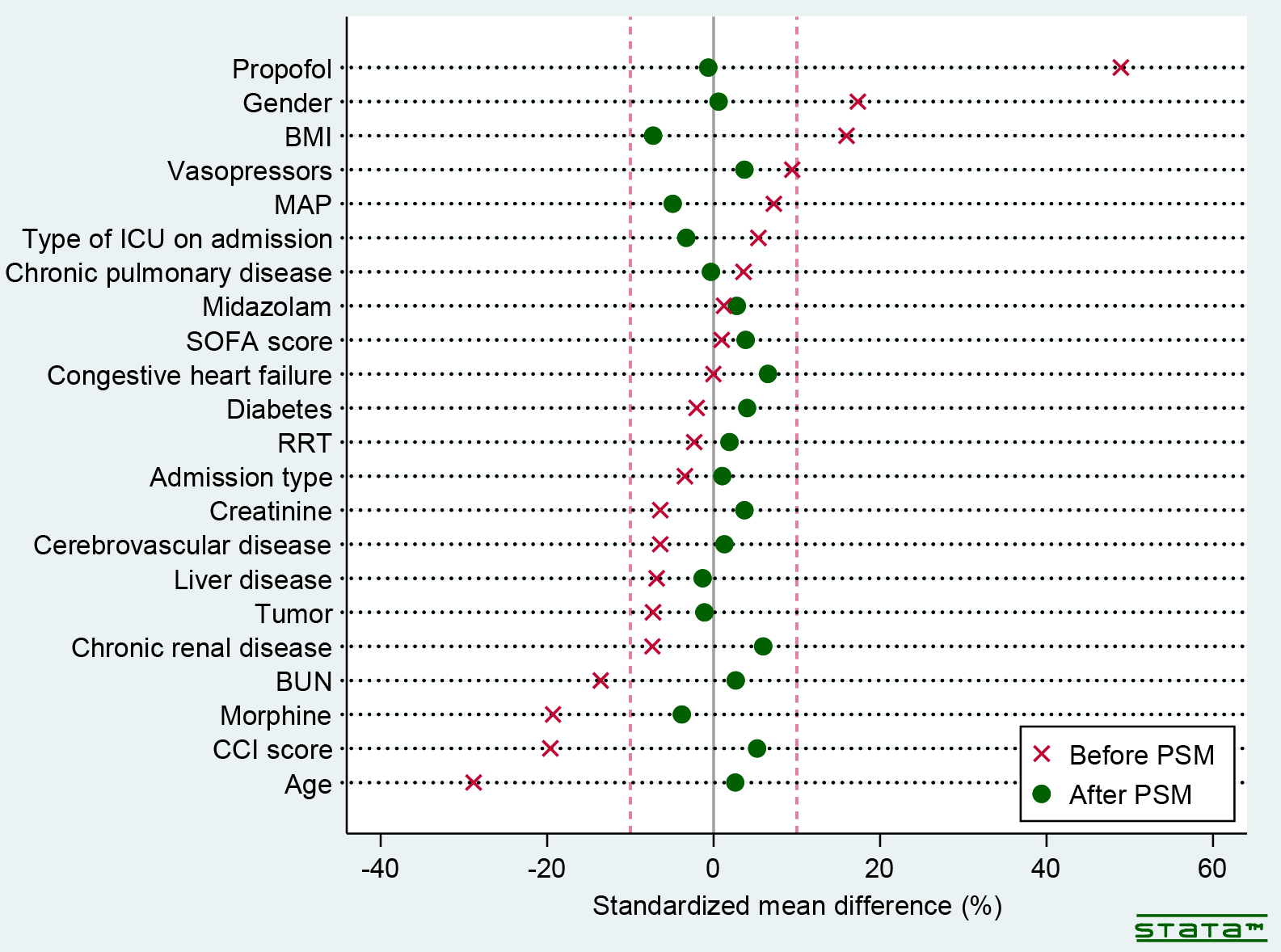


Abbreviations: PSM: propensity score matching; BMI: body mass index; BUN: blood urea nitrogen; MAP: mean arterial pressure; SOFA: Sequential Organ Failure Assessment; CCI: Charlson Comorbidities Index; RRT: renal replacement therapy; ICU: intensive care unit
